# Synergistic Effect of DIBOA and Verbascoside from *Acanthus mollis* Leaf on Tyrosinase Inhibition

**DOI:** 10.3390/ijms232113536

**Published:** 2022-11-04

**Authors:** Patrícia Matos, António Paranhos, Maria Teresa Batista, Artur Figueirinha

**Affiliations:** 1 Faculty of Pharmacy, University of Coimbra, 3000-548 Coimbra, Portugal; 2 LAQV, REQUIMTE, Faculty of Pharmacy, University of Coimbra, 3000-548 Coimbra, Portugal; 3 CIEPQPF, FCTUC, Department of Chemical Engineering, University of Coimbra, 3000-213 Coimbra, Portugal; 4 Center for Pharmaceutical Studies, Faculty of Pharmacy, University of Coimbra, 3000-548 Coimbra, Portugal

**Keywords:** synergism, mushroom tyrosinase, benzoxazinoids, phenylpropanoids

## Abstract

Overexpression of melanin contributes to darkening of plant and fruit tissues and skin hyperpigmentation, leading to melasma or age spots. Although melanin biosynthesis is complex and involves several steps, a single enzyme known as tyrosinase is key to regulating this process. The melanogenesis pathway is initiated by oxidation of the starting material l-tyrosine (or l-DOPA) to dopaquinone by tyrosinase; the resulting quinone then serves as a substrate for subsequent steps that eventually lead to production of melanin. Medicinal plants are considered a good source of tyrosinase inhibitors. This study investigated the tyrosinase inhibitory activity of *A. mollis* leaf extracts and their phytochemicals. Significant activity was verified in the ethanol extract –EEt (IC_50_ = 1.21 µg/mL). Additionally, a kinetic study showed that this tyrosinase inhibition occurs by DIBOA (2,4-dihydroxy-1,4-benzoxazin-3-one) and verbascoside contribution through a non-competitive reaction mechanism. A synergistic effect on tyrosinase inhibition was observed in the binary combination of the compounds. In conclusion, both EEt and a mixture of two of its phytochemicals can be effective tyrosinase inhibitors and can be used as a bleaching agent for cosmetic formulations in the future.

## 1. Introduction

Melanin is a macromolecular pigment in fungi, bacteria, plants, and animals. In human skin, this pigment is produced by melanocyte cells, melanosomes, present in the basal layer of the epidermis, through a physiological process called melanogenesis that involves a series of complex enzymatic and chemical processes, as well as extrinsic factors, such as chemicals and ultraviolet (UV) rays [1]. Melanin is a high-molecular-weight biopolymer derived from tyrosine that plays a crucial role in protecting against damage from skin exposure to UV rays, coloring the skin, hair, feathers, and pupils [2]. In plants, melanin is closely related to browning of fruits and vegetables [3]. In addition to absorbing UV sunlight, melanin can act as a free radical scavenger, thereby scavenging reactive oxygen species (ROS) [4]. However, abnormal melanin production involves many negative aspects of life. Recent pharmacological investigations have revealed that abnormal melanocyte metabolism and imbalance in tyrosinase activity are indirectly or directly responsible for several dermatoses. For example, functional mutations that cause melanin deficiency can result in type 1 oculocutaneous albinism, an autosomal recessive disorder characterized by absence of pigment in hair, skin, and eyes [5]. Overexpression of melanin can also cause hyperpigmentation in the epidermis (e.g., freckles, melasma, and senile lentigines), affecting aesthetics and significantly increasing the risk of skin cancer (melanoma) [6]. Therefore, melanin has been seen as a relatively specific biomarker and therapeutic target for melanoma lesions. Melanoma is an aggressive tumor that overexpresses tyrosinase, and it is estimated that, globally, it causes approximately 57,000 deaths per year [7], mainly due to multiple resistance to the currently available anticancer agents. Recently, epidemiological evidence has been found linking melanin and Parkinson’s disease, a neurodegenerative disorder associated with loss of dopaminergic neurons in the brain [8].

Although melanin biosynthesis is complex and involves several steps, so far, only three major tyrosine-like proteins have been identified as co-regulators of melanogenesis: tyrosinase (TYR), tyrosinase-related protein 1 (TYRP1), and tyrosinase-related protein 2. (TYRP2). Tyrosinase (an oxidoreductase, E.C. 1.14.18.1) is considered the rate-limiting enzyme of melanogenesis [9]. This enzyme is present throughout the body and has several biological roles. Chemically, tyrosinase catalyzes two distinct reactions: hydroxylation of monophenols and oxidation of *o*-diphenols and quinones. The first reaction is monophenolase because L-tyrosine is oxygenated to L-dihydroxyphenylalanine (L-DOPA). The second reaction, diphenolase, oxidizes L-DOPA to L-dopaquinone.

Inhibiting tyrosinase activity is an effective and essential way to prevent excessive melanin synthesis. For these reasons, tyrosinase inhibitors are widely used in the cosmetic and pharmaceutical industries as new depigmenting agents and in the food industry as food preservatives. It is also exciting in medicine since this enzyme correlates with neurodegenerative diseases. Tyrosinase inhibitors have received increasing attention from both natural and synthetic sources. Still, most of them are unsuitable for commercial use due to their low efficacy, off-flavors, cost of production, and safety concerns [10]. Developing new tyrosinase inhibitors with potent activity and lower side effects is undoubtedly needed. For example, arbutin, kojic acid, and hydroquinones are well-known tyrosinase inhibitors commonly used in skin-lightening cosmetics or anti-hyperpigmented agents. However, kojic acid is unstable during storage and causes dermatitis and cytotoxicity, while arbutin has potential cytotoxicity and hydroquinones have cytotoxic and mutagenic effects for mammalian cells [10].

Moreover, hydroquinone, the product of arbutin hydrolysis, can also cause contact dermatitis [11]. Accordingly, all these undesirable side effects limit their human use. Finding skin whitening agents free of toxicity is necessary, allowing us to use them in treating hyperpigmentation as a cosmetic or pharmaceutical product.

The trend toward herbal compounds has increased in recent years due to their low toxicity and better inhibitory activities, thus making them an essential bet for diseases related to excessive melanin production. 

There is growing interest in studies of phenolic compounds due to their potential health benefits. Flavonoids are the most reported compounds for tyrosinase inhibition. However, other phenolic compounds also show promising results, such as caffeoyl phenylethanoid glycoside and verbascoside [9]. This compound has been identified in the *Acanthus* species. *Acanthus mollis* is a species mainly used in Europe to treat skin diseases. In traditional medicine, aerial parts are employed for eczema, psoriasis, and wound healing treatment in the form of infusions or decoctions crushed or applied directly on the skin [12].

In this study, the objective was to evaluate the ability of *A. mollis* extracts and their major compounds to inhibit mushroom tyrosinase activity. Moreover, the inhibition kinetics of the major phytochemicals during dopachrome production will be investigated.

## 2. Results

### 2.1. HPLC–PDA and HPLC–PDA–ESI/MSn

**Figure 1:**
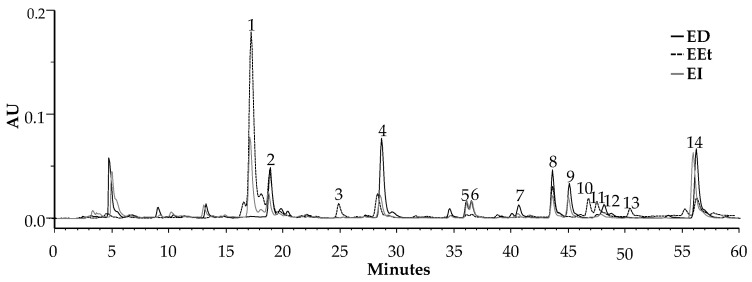
HPLC–PDA profile of the extracts from *A. mollis* leaves was recorded at 280 nm. ED—decoction; EEt—ethanol extract; EI—infusion.

Profiles from the ethanol (EEt) and two aqueous extracts, decoction (ED) and infusion (EI), obtained by HPLC–PDA, are illustrated in Figure 1. They are qualitatively similar, and their characteristic phytochemicals are phenolic acids, flavonoids, and benzoxazinoids. These classes of compounds have been identified based on their online UV spectra and according to literature data. The phenolic acids identified were phenylpropanoids [13]: flavonoids, which present maximum values at 272 nm (band II) and 335 nm (band I), were identified as apigenin derivatives [14], and benzoxazinoids by their characteristic spectra with a maximum of 254 nm and 280 nm and a shoulder at 286 nm [15].

Phytochemical characterization of the compounds was performed by HPLC–PDA–ESI/MS^n^ (Table 1). This table reports all the identified compounds, with their maximum UV absorption and MS fragmentation pattern. Identification was performed based on data available in the literature. 

Aqueous extracts present a chromatographic profile very similar to ethanolic extract; however, both the infusion and the decoction presented more flavonoids. Phenylpropanoids, as well as benzoxazinoids, are present in all the extracts.

Therefore, considering the previous identifications, the major compounds of the extracts were quantified (Table 2). Phenolic acids are particularly abundant in infusion and EEt, while benzoxazinoids occur the majority in EEt, DIBOA being broadly representative in ethanol extract, with approximately 5 g in 100 g of extract. In the infusion, the amount of this compound is significantly lower but higher than for the decoction since the temperature used to obtain this extract degrades the benzoxazinoid DIBOA, transforming it into its BOA derivative. Regarding verbascoside, it is verified that the infusion and decoction have similar amounts, slightly less than the ethanol extract.

Except for the decoction, the other extracts have higher amounts of DIBOA than verbascoside but in different proportions. The quantity of HBOA in the three extracts does not show relevant differences. The influence of the extractive method on the qualitative and mainly quantitative composition of the extracts is crucial for this species.

### 2.2. Antioxidant Activity

The study previously carried out by this group evaluated ethanol extract (Eet) for its antioxidant/antiradical capacity against DPPH. This extract, obtained from the leaves of *A. mollis*, showed antioxidant capacity for the DPPH assay, with an IC_50_ of 40.00 μg/mL. Considering the qualitative composition of the aqueous extracts, it was confirmed that, as with the ethanol extract, they also present a scavenging capacity for the DPPH radical, with an IC_50_ of 55.98 μg/mL and 45.05 μg/mL for the infusion and decoction, respectively. A direct correlation was verified between the concentration of verbascoside in the various extracts and their antioxidant activity.

### 2.3. Cytotoxicity Studies of the Extracts

The cytotoxicity of the extracts under investigation was evaluated based on the viability of the cells exposed to different concentrations. The results of the resazurin assay for keratinocyte (HaCaT) and fibroblast (3T3) cell lines with the extracts after 24 h of exposure are shown in Table 3. According to ISO 10993-5:2009 guidelines on the level of cytotoxicity, an extract is considered non-cytotoxic when cell viability is greater than 70% [24]. It is evident from the reported data that, for the HaCaT cell line, the infusion did not demonstrate cytotoxicity at any of the concentrations tested. In previously published results, the ethanol extract has not shown cytotoxicity for this cell line [25].

All the extracts do not present cytotoxicity for the fibroblasts at the concentrations tested.

Overall, the extracts showed weak to non-cytotoxic properties and, therefore, can be considered safe for use on human skin.

### 2.4. Tyrosinase Inhibitory Activity

The assay of mushroom tyrosinase inhibition of all extracts and their major compounds was performed. The ethanol extract showed better tyrosinase inhibition than the aqueous extracts, with an IC_50_ value of 1.21 ± 0.09 µg/mL. The aqueous extracts presented lower activity at the maximum concentration tested, 250 µg/mL, with 40% and 15% inhibition percentages for the infusion and decoction, respectively.

**Figure 2:**
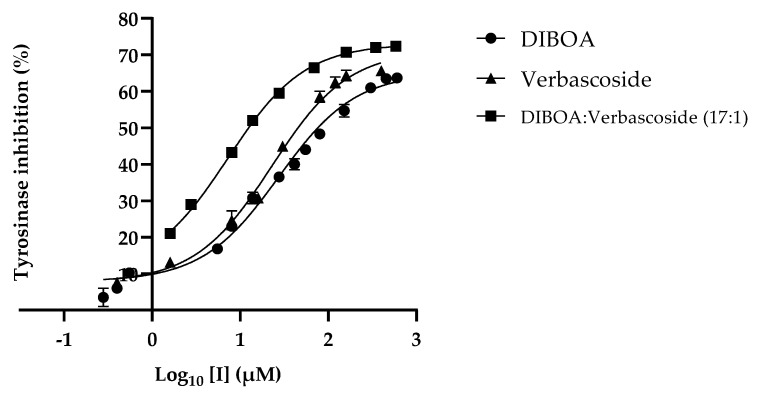
Inhibition plot of mushroom tyrosinase in the presence of the major compounds of the extracts.

Evaluation of the activity for the compounds DIBOA, HBOA, and verbascoside (Figure 2) showed that the best inhibitions were detected for the benzoxazinoid DIBOA and the phenylpropanoid verbascoside, with an IC_50_ of 27.24 ± 3.00 µM and 22.72 ± 2.83 µM, respectively. The HBOA did not present activity at higher concentrations tested, 1362.40 µM. 

Considering the concentration of the major compounds in the tested extracts and their tyrosinase inhibitory activity, this activity was assessed for a mixture of DIBOA and verbascoside at the same ratio current in the ethanol extract (17:1 µM). Under these conditions, the IC_50_ obtained was 7.23 ± 1.86 µM (Figure 2). This result may justify the contribution of these phytochemicals to the activity exhibited by the ethanol extract.

### 2.5. Kinetics Study of Tyrosinase

**Figure 3:**
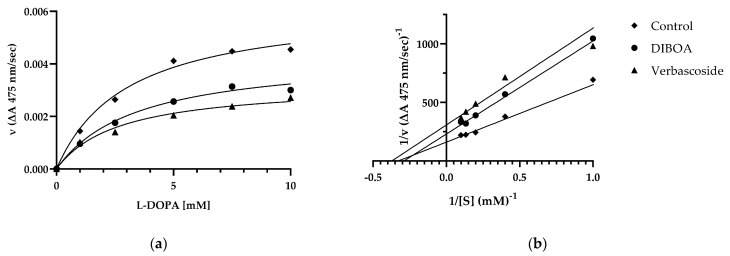
Michaelis–Menten (**a**) and Lineweaver–Burk (**b**) plot for inhibition of tyrosinase in the presence of DIBOA and verbascoside on tyrosinase with different substrate L-DOPA concentrations (1, 2.5, 5, 7.5, and 10 mM). Data are reported as mean ± SEM of n = 3 experiments.

Considering the IC_50_ values for the most active compounds, DIBOA and verbascoside, we performed a kinetic study to determine the type of inhibition on mushroom tyrosinase activity using Michaelis–Menten kinetics and Lineweaver–Burk plots. Increasing concentrations of the substrate L-DOPA were considered in the absence (control) or presence of each inhibitor (Figure 3a,b). The Lineweaver–Burk plot provided a family of straight lines intersecting simultaneously on the *X*-axis (Figure 3b).

The analysis showed that 1/V max presents different values, while the Km value of the enzymatic reaction remains the same at fixed concentrations of each inhibitor (Table 4). This behavior indicated that the compounds inhibit tyrosinase non-competitively from forming an enzyme inhibitor complex.

### 2.6. Synergism Determination in DIBOA and Verbascoside Combination

**Figure 4:**
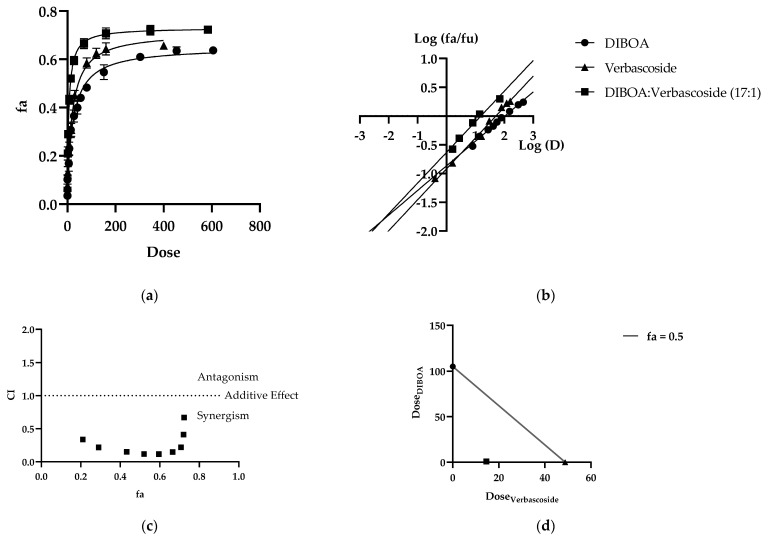
Combination analysis of DIBOA and verbascoside (**a**) dose–effect curve and its linearization with the (**b**) median effect plot for a single and combination treatment (**c**) combination index plot. (**d**) Dose isobologram at 50% effect combinations of DIBOA and verbascoside.

Given the inhibitory activity of the EEt extract, the significant compounds DIBOA and verbascoside were studied in combination for their mushroom tyrosinase inhibiting effect. The constant ratio combination based on Chou’s theory allows for dose–effect curves (Figure 4a), median effect plots (Figure 4b), CI plots (Figure 4c), and isobolograms (Figure 4d) of the dose reduction index (DRI) to reveal the type and extent of interactions between the compounds. It was observed that these methods offered complementary information and produced similar conclusions.

After performing the mushroom tyrosinase enzyme activity inhibition assay for each drug alone, dose–effect curves of each inhibitor alone and in binary combination were determined for calculation of parameters (Dm), (m), and (r). As shown in Table 5, all the drugs inhibited enzyme activity in a dose-dependent manner. The Dm value can be determined as the effective concentration needed to produce a 50% reduction in enzymatic activity. As shown in Table 5, the Dm values of DIBOA and verbascoside were 105.155 μM and 48.7695 μM, respectively. When combined, the Dm was less than the expected additive effect of each compound, indicating a substantial degree of synergy at an effective level of 50%. The median effect spots of all samples tested are shown in Figure 4b. All the (r) values of the curves were above 0.97, indicating acceptable compliance with the law of mass action.

Figure 4a shows the concentration-dependent enzyme inhibition of the individual and combined phytochemicals. In this case, we used a ratio of 17:1 (17 μM DIBOA and 1 μM verbascoside). The Dm (an indicator of drug potency) of DIBOA was 105.155 μM. In comparison, the Dm of verbascoside was 48.770 μM, derived from the median effect plots (Figure 4b). The combination of DIBOA with verbascoside reached a Dm value of 22.667, significantly decreasing the enzyme activity compared to treatment of the compounds alone. Using IC_50_ values, it was determined whether the interaction between DIBOA and verbascoside would be classified as synergistic (CI < 1), additive (CI = 1), or antagonistic (CI > 1). Chou and Talalay define the CI as the sum of the ratios between the combined dose of each drug and the individual dose to achieve a specific Fa. In our case, we derive CI = 0.22941 with Fa = 0.5. Figure 4c represents the rest of the CI vs. Fan. Over the entire range of effect levels of the compounds, interactions between them were universally synergistic at all effect levels (CI < 1), indicating that tyrosinase inhibition was markedly increased when DIBOA was combined with verbascoside at doses of IC_50_, as summarized in Table 5. The dose reduction index (DRI) also showed that the combination of DIBOA and verbascoside produced favorable dose reduction, demonstrating that a 50% decline in enzyme activity in a combination of the compounds decreased their concentrations by 4.91 and 38.73 for DIBOA and verbascoside, respectively, to obtain the same reduction in enzymatic activity.

Furthermore, the isobologram plot demonstrates the change in the combination of DIBOA and verbascoside doses to reach Fa = 0.5 (Figure 4d). At an effect level of less than 50% inhibition, the combination of the data points was located below the additivity line, indicating synergism.

## 3. Discussion

Tyrosinase inhibitors are chemical agents capable of reducing enzymatic reactions, such as browning of foods and melanogenesis of human skin. Therefore, these agents have good commercial potential in the food processing and cosmetics industries, where numerous studies have been developed. Many natural or synthetic compounds have been investigated for their ability to inhibit tyrosinase. Arbutin and ascorbic acid are referred to as tyrosinase inhibitors; however, they have some characteristics that limit them, namely their instability or even thermal sensitivity, which can lead to their decomposition [10]. Other compounds described as potent inhibitors show cytotoxic and mutagenic effects against mammalian cells. Hydroquinone is potentially mutagenic to mammalian cells and can cause contact dermatitis [26]. Another compound that has aroused great interest due to its potent inhibition is kojic acid [27]. This compound is frequently used in docking studies and was one of the first inhibitors to be identified. However, this compound has high carcinogenesis [28], which limits it to cosmetic uses. All these undesirable side effects restrict its human use. Therefore, it is necessary to find skin-lightening agents that are toxicity-free, allowing us to use them both cosmetically and in food, as well as medicinally, in treating hyperpigmentation, for example.

In this study, an ethanol extract of *A. mollis* leaf showed the best activity to inhibit mushroom tyrosinase, with an IC_50_ close to 1 µg/mL and without cytotoxicity to skin cells, such as keratinocytes and fibroblasts at active concentrations. These results suggest that this extract is safe for topical use in humans. Traditional medicine uses this plant for skin diseases [12].

In the genus *Acanthus*, studies of anti-tyrosinase activity are recent. In 2019, Gong and colleagues tested the ability of condensed tannins isolated from *A. ilicifolius* leaf to inhibit mushroom tyrosinase with an IC_50_ of approximately 20 µg/mL [29]. More recently, a correlation of this activity with the amount of verbascoside present in the methanol extract of *A. spinosus* was established [30]. A similar approach was referred to by Burgos and researchers, who isolated verbascoside from *A. mollis* leaf, obtaining an IC_50_ of about 84 µM for tyrosinase inhibitory activity [31]. In our work, higher activity was verified for the ethanol extract from *A. mollis* leaf, suggesting a contribution of other compounds to this activity. Too similar concentrations of verbascoside in the three studied extracts and significant differences in the DIBOA amounts were registered. A direct correlation between DIBOA concentration in the three extracts and tyrosinase inhibitory activity was verified. A standard of this benzoxazinoid presented an IC_50_ very close to that of verbascoside, with about 27 µM. Therefore, this compound is referred to as a possible tyrosinase inhibitor for the first time, thus becoming a promising compound in further studies.

In pharmacology, there is a growing concern in studying the synergistic or antagonistic potential when molecules are combined. However, studies on mixtures of phytochemicals have not been explored for inhibition of tyrosinase activity, so there is little information about the synergistic effects of the inhibitors. Our study aimed to provide experimental results based on in vitro combination experiments. According to the results of our research, the DIBOA and verbascoside, when tested in the proportion in which they are found in the ethanol extract, have an IC_50_ of about 7 µM, thus implying that the combination of the two phytochemicals has a synergistic effect, as we demonstrated through the Chou–Talalay method [32]. Therefore, it may be helpful as a guide to selecting a particular combination to be subjected to extensive in vivo studies and subsequent clinical trials.

Moreover, our results showed promising binary synergistic combinations with favorable DRI, which could be valuable for regimens against tyrosinase activity. In 2007, Sené and his collaborators patented verbascoside with the flavonoid luteolin or any extract that had a mixture of the two in cosmetic or pharmaceutical use for hyperpigmentation [33]. These researchers found a synergistic effect of these compounds once present in the extract of *Buddleja axillaris*. These results demonstrate the importance of researching the combination of molecules with a view to the synergistic effects.

Some of the undesired effects many molecules have when they inhibit the enzyme is their instability in staying bound. Therefore, it was essential to investigate the compound–enzyme interactions in this work. In a kinetic study, we can verify that both DIBOA and verbascoside inhibit the enzyme in a non-competitive way, implying that they bind to the enzyme–substrate complex. In that sense, our compounds that bind in a non-competitive way are an asset.

To be associated with the tyrosinase inhibitor effect, there is also to consider the antioxidant and anti-inflammatory activities of the ethanol extract of *Acanthus mollis* leaf and its phytochemicals, verbascoside and DIBOA [25]. The skin is the largest organ in the human body. It can suffer damage caused by free radicals, such as reactive oxygen species (ROS), that can lead to premature aging, inflammation, or even skin cancer. This study verified a direct correlation between the concentration of verbascoside in the extracts and their antioxidant activity. Critical applications have been described for this phenylpropanoid, including as a topical photoprotective and in chemoprevention of UV-induced non-melanoma skin cancer [31,32]. Moreover, Sperotto and collaborators demonstrated that this compound promotes skin repair and improves skin inflammation due to its ROS scavenging activity, inducing antioxidant, iron chelator, and glutathione transferase (GST) [33].

Therefore, *A. mollis* ethanol extract and its majority compounds can also promote skin repair and suppression of inflammatory reactions, supporting the use of this plant in traditional medicine for skin diseases. 

## 4. Materials and Methods

### 4.1. Material of Study

Standards of 2,4-dihydroxy-1,4-benzoxazin-3-one, DIBOA (181.15 g/mol, 91% purity); 2-hydroxy-1,4-benzoxazin-3-one, HBOA (165.15 g/mol, 95% purity) obtained from Enanine Ltd. (Kyiv, Ukraine); and 2-(3,4-dihydroxyphenyl)ethyl-1-*O*-*α*-l-rhamnopyranosyl-(1→3)-(4-*O*-Ε-caffeoyl)-*β*-d-glucopyranoside, verbascoside (624.6 g/mol) from Extrasynthese (Lyon, France) were used. 

*A. mollis* L. leaf was obtained from Coimbra city (40°12′20″ N, 8°25′22″ W) in February 2018. A voucher specimen (A. Figueirinha 01015) was deposited at the Herbarium of Medicinal Plants, Faculty of Pharmacy, University of Coimbra.

The plant material was kept refrigerated at −20 °C, away from light and moisture, until used.

### 4.2. Preparation of Extracts

*A. mollis* L. leaves were lyophilized, milled, and sieved (2500 meshes/cm^2^). Three extracts were prepared from the powdered plant (pp).

#### 4.2.1. Ethanol Extract

(EEt)—pp (4 g) was extracted with 96% ethanol (200 mL) using an electromagnetic stirrer for one hour. After filtration, water was added, and the extract was placed overnight in the cold and centrifuged to remove chlorophyll. The supernatant has been reserved. 

#### 4.2.2. Infusion

(EI)—pp (1 g) was placed in an infusion mug, to which boiling water (100 mL) was added and left to rest for 10 min. Afterward, centrifugation and subsequent concentration in a rotary evaporator, followed by lyophilization, were carried out. 

#### 4.2.3. Decoction

(ED)—pp (1 g) was added to water (100 mL) and boiled for 15 min. Subsequently, the same procedure for the infusion was used.

### 4.3. High-Performance Liquid Chromatography with Photodiode Array Detection (HPLC–PDA)

Phytochemical evaluation was performed using an HPLC–PDA Gilson equipped with two pumps (models 305 and 306), a mixer (Model 811 B), a manometric module (model 805), and an autosampler (Gilson 234 autoinjector) coupled to a PDA (Gilson model 170) and a control station Unipoint System data (Unipoint^®^ 2.10). The analyses were carried out on a Waters^®^ RP18 Spherisorb ODS-2 column (250 × 4.6 mm; 5 μm particle size) maintained at 35 °C and protected by a guard column KS 30/4 Nucleosil 120–5 C-18, Macherey-Nagel (Duren, Germany). The mobile phase consisted of a 5% aqueous formic acid solution (A) and acetonitrile (B), used at a flow rate of 1 mL/min. The gradient was 0–100% B (0–75 min) and isocratic for 15 min. The UV-V profiles were acquired in the 200–600 nm range, and chromatograms were recorded at 280 and 320 nm. Identification has been confirmed with the standards DIBOA, HBOA, and verbascoside, as well as by the characteristic shape of the online UV spectra of the benzoxazinoids, phenylpropanoids, and apigenin derivatives [13,14,15].

Detection and quantification limits (LOD and LOQ) of benzoxazinoids and verbascoside were determined from the parameters of the calibration curves represented in Table 6. Three independent injections were performed for each sample, injecting 100 μL of extract and standards dissolved in water and microfiltered.

### 4.4. HPLC–PDA–ESI/MS^n^ Analysis

Chromatographic profiles were characterized using a Liquid Chromatograph of High Performance (Finnigan Surveyor, THERMO, Waltham, MA, EUA) coupled to a Diode Array Spectrophotometer (Finnigan Surveyor, THERMO) and a Linear Ion Trap Mass Spectrometer (LIT-MS) (LTQ XL, Thermo Scientific, Waltham, MA, EUA).

The LC column (Waters Spherisorb ODS2; 3 μm, 150 × 2.1 mm) was preceded by a guard cartridge (Waters Spherisorb ODS2; 5 μm, 10 × 4.6 mm), and separation was carried out at 20 °C. The mobile phase consisted of 2% aqueous formic acid (solvent A) and methanol (solvent B). The gradient profile used was 0–10 min, 5–15% B; 10–15 min, 15–25% B; 15–40 min, 25–50% B; 40–50 min, 50–80% B, at a flow rate of 200 μL/min. 

The first detection was made in the diode array spectrophotometer in a wavelength range of 200–500 nm using 280 and 320 nm as preferred wavelengths. The second detection was created with the mass spectrometer. It was operated in negative electrospray ionization (ESI) mode and programmed to perform a series of three scans: a total mass (MS) and an MS^2^ and MS^3^ of the most abundant ion. The collision gas was helium, with a normalized collision energy of 35%. Nitrogen was used as nebulizing gas, with a sheath gas flow of 20 (arbitrary unit) and the auxiliary gas flow of 5 (arbitrary unit). Capillary temperature and voltage were set at 275 °C and −25.00 V, respectively. The source voltage was set at 5.00 kV.

### 4.5. Antioxidant Activity

Antioxidant activity was evaluated using the 2,2-diphenyl-1-picrylhydrazyl (DPPH) radical method described by Blois (1958) [34] and according to previous studies performed in this laboratory [25]. The results were expressed in IC_50_ values, indicating the concentrations of samples required for a 50% reduction in the absorbance of the DPPH solution.

### 4.6. In Vitro Cytotoxicity Evaluation

#### 4.6.1. Cell Culture

Two different cell lines were used to assess the potential cytotoxicity of the extracts: the fibroblasts cell line (3T3) and the human keratinocytes cell line (HaCaT) obtained from DKFZ (Heidelberg) and kindly supplied by Dr. Eugenia Carvalho (Center for Neurosciences and Cell Biology, University of Coimbra, Portugal). Keratinocytes and fibroblasts were cultured in Dulbecco’s Modified Eagle Medium (high glucose) supplemented with 10% heat-inactivated fetal bovine serum, 3.7 g/L sodium bicarbonate, 25 mM glucose, 100 U/mL penicillin, and 100 μg/mL streptomycin at 37 °C in a humidified atmosphere of 95% air and 5% CO_2_. The experiments monitored the cells under an optical microscope to detect any morphological change.

#### 4.6.2. Assessment of Cell Viability by Resazurin Assay

Resazurin assay was used to evaluate the cell viability, which is based on the ability of live cells to convert resazurin (blue nonfluorescent) into resorufin (fluorescent pink dye) [35]. Therefore, dye reduction magnitude correlates with number of viable cells. Therefore, after the treatment described above, fibroblasts and keratinocytes were incubated with a resazurin solution (50 μM in culture medium) for two hours at 37 °C in a humidified atmosphere of 5% CO_2_ and 95% air. Quantification of resorufin was performed in an ELISA microplate reader (Biotek Synergy HT) at 570 nm using a 620 nm reference filter. A cell-free control was performed to exclude the nonspecific effects of the extracts on resazurin.

### 4.7. Mushroom Tyrosinase Inhibitory Effect of A. mollis Extracts and Its Phytochemicals

The tyrosinase activity assay was performed with some modifications in the procedure described by Masamoto (2003) [36] to determine the inhibitory effects of the extracts, as well as the major compounds present in them. The reaction mixture containing L-DOPA (Sigma-Aldrich, St. Louis, MO, USA) (0.5 mM) in 0.1 M phosphate buffer at pH 6.8 with or without sample in a total volume of 1 mL was pre-incubated at 25 °C for 5 min. Mushroom tyrosinase (EC 1.14.18.1, Sigma-Aldrich, St. Louis, MO, USA) buffer solution (500 U/mL) (0.1 mL) was added, and the reaction was monitored, with the change in absorbance at 475 nm for 120 s at 10-sec intervals in a UV–Vis spectrophotometer—Jasco V-530. Enzyme activity in the absence of inhibitors was defined as 100%. The half-maximal inhibitory concentration (IC_50_) was estimated by the plot of relative enzymatic inhibition vs. logarithm of inhibitor concentration.

### 4.8. Kinetic Analysis of Tyrosinase Inhibition

The type of enzyme inhibition exerted by DIBOA and verbascoside at concentrations near the IC_50_ was evaluated from kinetic studies using different substrate concentrations (1–10 mM L-DOPA) applying Michaelis–Menten and Lineweaver–Burk plots. The Michaelis–Menten equation can be written in usual form:

(1) $$v=\frac{V_{máx}\;\left[S\right]}{K_{m}+\left[S\right]}$$

where v is the rate of reaction in the presence or absence of an inhibitor, $V_{máx}$ is the maximal reaction rate, $K_{m}$ is the Michaelis–Menten constant, and [*S*] is that of the substrate. To obtain the Lineweaver–Burk equation, the Michaelis–Menten equation was transformed in the form of:

(2) $$\frac{1}{v}=\frac{K_{m}}{V_{máx}}\frac{1}{\left[S\right]}+\frac{1}{V_{máx}}$$

### 4.9. Study of the Synergistic Inhibitory Activity on the Mushroom Tyrosinase Enzyme

#### 4.9.1. Combination Effects Determined by the “Fixed Ratio” Method

DIBOA and verbascoside were tested in a fixed ratio combination considering each proportion in the ethanol extract to assess their combined effects on tyrosinase inhibition. Several published methodologies, including median effect analysis, isobologram, combination index, and dose reduction index analysis, were applied to assess the nature of the combination proposed in this study.

##### Median Effect Analysis

Using the Chou–Talalay method [32], the sigmoidal dose–effect curve for every single agent and its binary combination was easily plotted and then transformed into their corresponding linear median effect plots based on the median effect equation.

The median effect equation suggested by Chou (2006) [37] can be broken down as follows:

(3) $$\frac{fa}{fu}={\left(\frac{D}{Dm}\right)}^{m}$$

where *D* is the dose of a drug, *Dm* is the median effect dose that decreases enzyme activity by 50%, fa is the fraction affected by *D* (i.e., percent effect/100), and fu is the unaffected fraction (*fu* = 1 − *fa*); m is the slope of the median effect plot that denotes the shape that the dose–effect curve can be rearranged into:

(4) $$\log\left(\frac{fa}{fu}\right)=m\log\left(D\right)-m\log\left(Dm\right)$$

In the median effect plot, y = log(*fa*/*fu*) versus x = log(*D*); log (*Dm*) is the x-intercept.

##### Isobolographic Analysis

Dose isobolograms were generated to provide a fundamental basis for illustrating the dose-dependent interaction of drugs combined at various levels of effect. The combination in effect may be greater, equal to, or less than would be expected from individual agents. The isobologram is a two-coordinate graph, with each coordinate representing the concentration of drugs A and B, respectively. When applied as single drugs, the concentrations of drugs A and B needed to produce a particular effect (IC_50_, A and IC_50_, B when x = 50%) were placed at the x and y coordinates, respectively. The diagonal line of additivity was created connecting these two points ((IC_50_, A, 0) and (0, IC_50_, B) for a 50% effect isobologram plot). Then, the concentrations of A and B, which were used in the combination study to provide the same effect x (50%), were plotted on the same graph as a by point (CA,x, CB,x). The combination data points above or below the additivity line indicated antagonism or synergy, respectively.

##### Combination Index Analysis

The isobologram combination index (*CI*) equation that merges with the median effect equation provides a quantitative measure of the extent of combined drug interaction at a series of effect levels [38]. Numerical values of *CI* were calculated according to the following formula:

(5) $$CI=\frac{D_{1}}{{\left(Dx\right)}_{1}}+\frac{D_{2}}{{\left(Dx\right)}_{2}}$$

where (*Dx*)_1_ and (*Dx*)_2_ are the doses of each compound required to produce the level of enzymatic-activity-reducing effect, and *D*_1_ and *D*_2_ are the doses of the two compounds in combination that have the same effect. The CI was used to assess whether the combinations in effect produce synergistic (*CI* < 0.9), additive (*CI* = 0.9–1.1), or antagonistic (*CI* > 1.1) effect. The fa–*CI* graph represents an effects-oriented graph that displays the type of interaction, synergism, antagonism, or additive effect depending on the level of effect or potency (fa) of certain combined drugs on the enzyme. It is worth noting that the effect-oriented plot (fa-*CI*) and the dose-oriented isobologram provide an identical conclusion of synergism or antagonism.

##### Analysis of the Dose Reduction Index (DRI)

The dose reduction index (DRI) of combinations of two compounds indicates how often the dose can be reduced to achieve an effect comparable to that achieved by each compound alone. A change in DRI > 1 is helpful as it implies a reduction in the doses of the combined agents while maintaining efficacy.

### 4.10. Statistical Analysis

The results were expressed as mean ± standard error of the mean (SEM) of at least three independent experiments (n ≥ 3).

Statistical analysis was performed using GraphPad (Dr. Harvey Motulsky, San Diego, CA, USA), version 9.0.0, and was analyzed at a 5% significance level. To analyze the different concentrations, one-way ANOVA was used, followed by Dunnett’s post hoc or nonparametric tests, namely the Kruskal–Wallis test with Dunn’s post hoc test, when justified, adjusted for multiple comparisons if there were three or more independent groups in the analysis. The level of significance was *p* < 0.05.

## 5. Conclusions

An ethanol extract from the *Acanthus mollis* leaf showed significant activity for tyrosinase inhibition (1.21 µg/mL), which was substantially higher than the aqueous extracts. Its main phytochemicals, namely DIBOA and verbascoside, also showed tyrosinase inhibitory activity. The individual compounds exhibited a non-competitive inhibitor–enzyme interaction. A synergic effect was verified with a mixture of these two compounds in the same proportion as ethanol extract. 

Tyrosinase inhibitors are chemical agents capable of reducing enzymatic reactions, such as browning of foods and melanogenesis of human skin. Therefore, they have good commercial potential in the food processing, cosmetic, and pharmaceutical industries.

The safety of the *Acanthus mollis* leaf ethanol extract to the keratinocyte and fibroblast cell lines and its anti-tyrosinase activity constitute promising results and encourage additional future studies, which are essential to investigate its effects at the cellular level.

## Figures and Tables

**Table 1:** Benzoxazinoids and phenolic compounds were identified in the extracts by HPLC–PDA–ESI/MS^n^.

No.	λ_máx_ (nm)	[M-H]^-^	HPLC–ESI/MSn m/z	Tentative Identification	Compounds Detected		
					EEt	EI	ED
1	256, 264 sh, 280, 287 sh	-	-	DIBOA *	✓	✓	✓
2	255, 277, 284 sh	-	-	HBOA *	✓	✓	✓
3	261, 283 sh, 321 sh, 329	**387**	MS^2^: 163; **207**; 369 MS^3^: **163**	**Medioresinol**	✓	✗	✗
4	271, 277 sh	-	-	**BOA**	✓	✓	✓
5	256, 287 sh, 300 sh, 330	**639**	MS^2^: 529; **621** MS^3^: **459**; 469	***β*-OH-verbascoside**	✓	✓	✓
6	256, 285 sh, 300 sh, 331	**639**	MS^2^: 529; **621** MS^3^: **459**; 469	***β*-OH-verbascoside**	✓	✓	✓
7	267, 336	**621**	MS^2^: **351** MS^3^: 113; 175; **193**; 289; 333	**Apigenin di-glucuronide**	✗	✓	✓
8	258, 288 sh, 291, 299 sh, 331	**623**	MS^2^: **461** MS^3^: **315**	Verbascoside *	✓	✓	✓
9	260, 268 sh, 287 sh, 342	**637**	MS^2^: 285; **351** MS^3^: 113; 175; **193**; 289; 333	**Luteolin di-glucuronide**	✗	✓	✓
10	248, 288 sh, 300 sh, 331	**667**	MS^2^: **621** MS^3^: 179; **459**	***β*-EtOH-verbascoside isomer**	✓	✗	✗
11	247, 289 sh, 300 sh, 330	**667**	MS^2^: **621** MS^3^: 179; **459**; 469	***β*-EtOH-verbascoside isomer**	✓	✗	✗
12	267, 289 sh, 342	**651**	MS^2^: **351**; 517 MS^3^: 113; 175; **193**; 289; 333	**Hispidulin di-glucuronide**	✗	✗	✓
13	261, 286, 299, 331	**623**	MS^2^: **461** MS^3^: 135; 161; 297; **315**	**Verbascoside isomer**	✓	✗	✗
14	272, 334	**475**	MS^2^: **299**; 175 MS^3^: **284**	**Hispidulin glucuronide**	✓	✓	✓

According to the authors, identification was based on the UV–Vis spectra, molecular weight, and fragmentation patterns [16,17,18,19,20,21,22,23]. EI—infusion; ED—decoction; EEt—ethanol extract; sh—shoulder; bold—base peaks in MS spectra; * identification confirmed by comparison with authentic standards; DIBOA (2,4-dihydroxy-1,4-benzoxazin-3-one); HBOA (2-hydroxy-1,4-benzoxazin-3-one); BOA (benzoxazoline-2-on.e).

**Table 2:** Quantification of the benzoxazinoids and verbascoside in the extracts by HPLC–PDA.

No.	Compounds	EEt	EI	ED
		g of Compound/100 g of Extract ^1^		
1	DIBOA	4.77 ± 0.11	2.18 ± 0.04	<LOQ
2	HBOA	0.78 ± 0.10	0.41 ± 0.04	0.64 ± 0.04
5	*β*-OH-verbascoside	<LOD	<LOQ	<LOQ
6	*β*-OH-verbascoside	<LOD	0.42 ± 0.05	<LOQ
8	Verbascoside	1.02 ± 0.13	0.61 ± 0.05	0.77 ± 0.05
9	Luteolin di-glucuronide	---	<LOQ	<LOQ
10	*β*-EtOH-verbascoside isomer	<LOQ	---	---
11	*β*-EtOH-verbascoside isomer	<LOQ	---	---
12	Hispidulin di-glucuronide	---	---	<LOQ
13	Verbascoside isomer	<LOD	<LOQ	<LOQ
14	Hispidulin glucuronide	<LOQ	0.52 ± 0.02	0.30 ± 0.02

^1^ Values are the mean ± standard deviation of three replicates; LOD (limit of detection); LOQ (limit of quantification); EEt—ethanol extract; EI—infusion; ED—decoction.

**Table 3:** Cytotoxicity of the extracts on keratinocytes (HaCat) and fibroblasts (3T3).

	Concentration, µg/mL	HaCaT ^1^	3T3 ^1^
Control	0	100.00	100.00
Eet	15	100.00 ± 2.08	n.d.
	30	98.67 ± 5.21	86.50 ± 1.81
	60	96.33 ± 4.81	79.50 ± 2.18 **
	120	87.00 ± 5.86	78.83 ± 2.32 **
	180	n.d.	77.50 ± 2.35 ***
	240	78.67 ± 4.10	76.50 ± 2.15 ***
EI	15	97.00 ± 1.00	n.d.
	30	98.33 ± 1.20	94.50 ± 192
	60	101.3 ± 4.98	96.58 ± 2.35
	120	102.3 ± 0.33	90.67 ± 1.88 *
	180	n.d.	86.33 ± 1.48 ***
	240	105.7 ± 5.953	84.92 ± 1.69 ***
ED	30	n.d.	91.42 ± 2.87
	60		88.67 ± 4.77
	120		84.17 ± 5.11 *
	180		85.83 ± 4.65 *
	240		89.92 ± 5.14

^1^ Values are mean ± SEM of three replicates; n.d.—not determined; (*** *p* ≤ 0.001; ** *p* ≤ 0.001 * *p* ≤ 0.05, versus control cells). Eet—ethanol extract; EI—infusion; ED—decoction.

**Table 4:** Kinetic parameters of the mushroom tyrosinase for oxidation of L-DOPA in inhibitor DIBOA and verbascoside at concentrations 27 µM and 23 µM, respectively.

Samples	V_máx_ (∆A/s)	K_m_ (mM)	Inhibition Type
Control	6.22 × 10^−3^	3.05	Non-competitive
DIBOA 27 µM	4.36 × 10^−3^	3.46	
Verbascoside 23 µM	3.26 × 10^−3^	2.69	

**Table 5:** Data summary of the dose–effect curve and Chou–Talalay parameters of binary drug combinations against tyrosinase enzyme activity.

Samples	Dm (μM)	m	r	CI at fa = 0.5	DRI
DIBOA	105.155	0.42845	0.99545	-	4.91197
Verbascoside	48.7695	0.53767	0.99768	-	38.7278
DIBOA:Verbascoside (17:1)	22.6672	0.38602	0.96649	0.22941	-

**Table 6:** Linearity, the limit of detection (LOD), and the limit of quantification (LOQ) of the standard compounds used as reference.

Standard Compound	Range Concentration (µg/mL)	n ^1^	Slope	Intercept	R^2^	LOD (µg/mL)	LOQ (µg/mL)
DIBOA	4.55–228	5	1.86 × 10^6^	1.37 × 10^7^	0.9994	0.41 ± 1.51	3.47 ± 1.49
HBOA	4.75–238	5	2.72 × 10^6^	1.98 × 10^6^	0.9996	2.05 ± 1.28	8.56 ± 1.26
Verbascoside	0.78–500	5	1.99 × 10^6^	−1.23 × 10^6^	0.9999	3.87 ± 1.61	11.4 ± 0.16
Apigenin	0.5–100	5	5.87 × 10^6^	−5.17 × 10^6^	0.9993	2.42 ± 0.72	6.01 ± 0.71

^1^ Number of points used for regression of standard solutions. Injections were completed in duplicate.

## Data Availability

The data present in this current study are available from the corresponding author upon reasonable request.
